# Crowd-Sourced Amputee Gait Data: A Feasibility Study Using YouTube Videos of Unilateral Trans-Femoral Gait

**DOI:** 10.1371/journal.pone.0165287

**Published:** 2016-10-20

**Authors:** James Gardiner, Nuwan Gunarathne, David Howard, Laurence Kenney

**Affiliations:** 1 School of Computing, Science and Engineering, University of Salford, Salford, United Kingdom; 2 School of Health Sciences, University of Salford, Salford, United Kingdom; Northwestern University, UNITED STATES

## Abstract

Collecting large datasets of amputee gait data is notoriously difficult. Additionally, collecting data on less prevalent amputations or on gait activities other than level walking and running on hard surfaces is rarely attempted. However, with the wealth of user-generated content on the Internet, the scope for collecting amputee gait data from alternative sources other than traditional gait labs is intriguing. Here we investigate the potential of YouTube videos to provide gait data on amputee walking. We use an example dataset of trans-femoral amputees level walking at self-selected speeds to collect temporal gait parameters and calculate gait asymmetry. We compare our YouTube data with typical literature values, and show that our methodology produces results that are highly comparable to data collected in a traditional manner. The similarity between the results of our novel methodology and literature values lends confidence to our technique. Nevertheless, clear challenges with the collection and interpretation of crowd-sourced gait data remain, including long term access to datasets, and a lack of validity and reliability studies in this area.

## Introduction

Collecting large datasets of amputee gait is notoriously difficult. Especially, for less prevalent amputation levels and for elderly vascular amputees (aged over 65), the largest and most challenging to rehabilitate section of the amputee population [1]. Indeed, due to the practicalities of collecting kinematic and kinetic data many studies of amputee gait performance are conducted in highly controlled laboratory environment and include small numbers of subjects. For example Hofstad et al.’s Cochrane review [2] of prosthetic ankle/feet joints includes 26 studies, with an average of just over 9 patients per study, with only 5 studies including more than 10 participants. Additionally, although a few recent studies have begun to explore amputee walking on surfaces representative of the everyday environment [3–7] many, if not most, amputee gait studies are conducted in gait laboratories, which greatly limits the extent to which results can be generalised. This is especially important, since walking in the community involves many surfaces that are not level, smooth or flat, for example, stairs, ramps, gravel paths, grass etc. Nevertheless, the gait laboratory approach has provided detailed insights into the relationships between prosthesis properties, amputee characteristics and gait, and will of course continue to play a central role in research for the foreseeable future. However, larger datasets on the performance of subjects with more unusual amputation levels and/or challenging pathologies, and datasets on conditions outside of typical lab environments are scarcer in current literature and remain difficult to collect. In this manuscript we investigate the potential of ‘crowd-source’ video footage as a source of amputee gait data to help address these issues, and provide substantial datasets of high quality amputee gait data.

Crowd-sourcing is the idea of outsourcing data collection/processing to the general public and has been gaining popularity in the scientific community. Indeed, several high profile studies have caught the public imagination [8] such as protein folding game ‘foldit’ and galaxy classification website ‘galaxy zoo’. These projects, however, require active participation from members of the public. There is also a wealth of user generated content on the Internet that requires no further input from the public and is potentially a valuable source of data for researchers. Indeed, many websites exist solely to promote user-generated content, such as photos, music and videos. YouTube in particular has been used as a tool to study a variety of medical topics such as kidney stones [9], cardiopulmonary resuscitation [10] and Human Papillomavirus (HPV) vaccination [11].

Many amputees post videos of themselves using their prosthesis in a variety of scenarios, such as running, walking and stair climbing. However, as far the authors are aware the potential of these crowd-sourced videos for amputee gait analysis has yet to be acknowledged or tested. Here we investigate the potential of user-generated videos (from websites such as YouTube) to provide temporal gait parameters for amputees using their prosthetic devices. To allow for validation of our results against existing literature, we focus our analysis on an example dataset of unilateral trans-femoral amputees level walking at a self-selected speed and compare our data against published values.

## Materials and Methods

The video sharing website YouTube (www.youtube.com) was searched for videos of trans-femoral amputee gait. The searches of YouTube’s database were conducted between the 18^th^ of February and the 31^st^ March 2015 by a single researcher (N.G.). The website was queried using the following terms in a variety of combinations: amputee, walking, prosthetics, gait, trans-femoral. Only the first page of search results (typically 20 results per page) was checked for videos, due to that fact that any search in YouTube typically produces tens of thousands of results. Videos that contained clear footage of adult unilateral trans-femoral amputee walking on level ground were downloaded for further analysis, regardless of language. Videos were only selected for analysis where both legs were clearly visible and walking was in a straight line at a consistent walking speed for a minimum of five complete gait cycles. Both company promotional videos and individual amputee’s own videos were included. Videos that clearly contained early rehabilitation footage were rejected, since it was thought that these videos might skew the results. All data from the YouTube videos were anonymised and collected under the ‘Fair Use’ and ‘Exceptions to Copyright’ rules for non-commercial research. Please see: https://www.youtube.com/yt/copyright/en-GB/fair-use.html and https://www.gov.uk/guidance/exceptions-to-copyright.

The downloaded videos were analysed using Tracker 4.87 (OpenSourcePhysics), which allows the user to process the YouTube footage frame by frame. Therefore, the timings of frames in which the heel first visibly touched the ground were recorded as the heel strikes. Likewise the timings of the video frames in which the toe first visibly left the ground were recorded as toe-off. This process was repeated for both legs for five complete gait cycles and the collected data was used to calculate the gait cycle time, and also the swing and stance times for both the prosthetic and sound leg. The five complete gait cycles were averaged to give mean values of these temporal parameters. This temporal data was then used to calculate the duty factors (i.e. stance phase as a percentage of the gait cycle) for the prosthetic and sound leg. To test whether the data we gathered was significantly asymmetric, a commonly used gait asymmetry index [12–15] was calculated from the stance phase data using the following equation

$$A=\frac{S_{i}-S_{p}}{\frac{1}{2}\left(S_{i}+S_{p}\right)}$$

where *A* is asymmetry index, *S*_*i*_ is intact stance phase duration and *S*_*p*_ is prosthetic stance phase duration. The asymmetry index was tested using a two way t-test with an asymmetry index of zero being the null hypothesis representing perfectly symmetrical gait.

Our temporal amputee gait data was then compared with existing data from the literature [14–18] to assess the variability of our data, and hence the feasibility of our methodology for future research studies. We assumed that all the studies (our research and the literature) draw from the same overall population of unilateral trans-femoral amputees and therefore conducted an ANOVA on the results to test for significant differences between studies. A Tukey-Kramer post hoc test was conducted to identify which studies were significantly different. The ANOVA and Tukey-Kramer post hoc were performed using MATLAB^®^ R2014b with the statistics toolbox (The MathWorks, Inc., Natick, Massachusetts, United States). The study of van der Linden et al. [18] only included detailed results for a single amputee, therefore was not included in the ANOVA and was only included in the figures for visual reference.

## Results

Sixteen videos of adult unilateral trans-femoral level walking were downloaded from YouTube and used for further analysis. The detail of the study population can be seen in Table 1. Twelve of the videos contained footage of men walking and four of women. The majority of videos were of young or middle aged amputees using Ottobock Genium or C-legs, in trainers.

**Table 1:** Details of study population taken from the videos and associated online meta data.

Video	Sex	Age	Amputation reason	Time since amputation (years)	Leg	Footwear
1	M	Middle	Traumatic	8	Ottobock C-Leg	Trainers
2	M	Young	Unknown	<1	Ottobock 3R80	Trainers
3	F	Middle	Traumatic	1	Ottobock Genium	Sandals
4	M	Middle	Unknown	Unknown	Ottobock C-Leg	Trainers
5*	M	Unknown	Unknown	Unknown	Non-microprocessor	Unknown
6	F	Unclear	Unknown	Unknown	Ottobock C-Leg	Trainers
7	M	Young	Unknown	Unknown	Ottobock Genium	Trainers
8	M	Unclear	Unknown	Unknown	Ottobock Genium	Trainers
9	M	Young	Unknown	Unknown	Ottobock Genium	Trainers
10	M	Young	Unknown	Unknown	Ottobock Genium	Trainers
11	F	Middle	Bone cancer	>35	Ottobock C-Leg	Shoes
12	M	Young	Unknown	Unkown	Ottobock C-Leg	Trainers
13	M	Young	Congenital disorder	6	Ottobock C-Leg	Trainers
14	M	Young	Unknown	Unkown	Ottobock Genium	Trainers
15	M	Young	Unknown	Unkown	Ottobock C-Leg	Shoes/Trainers
16	F	Young	Traumatic	>3	Unknown	Shoes

* Video 5 was no longer online to allow additional information to be collected for the details of study population (suggestion by draft manuscript reviewer). Therefore only limited information was recorded for this video and this issue highlights the transient nature of data sources such as YouTube

**Fig 1:**
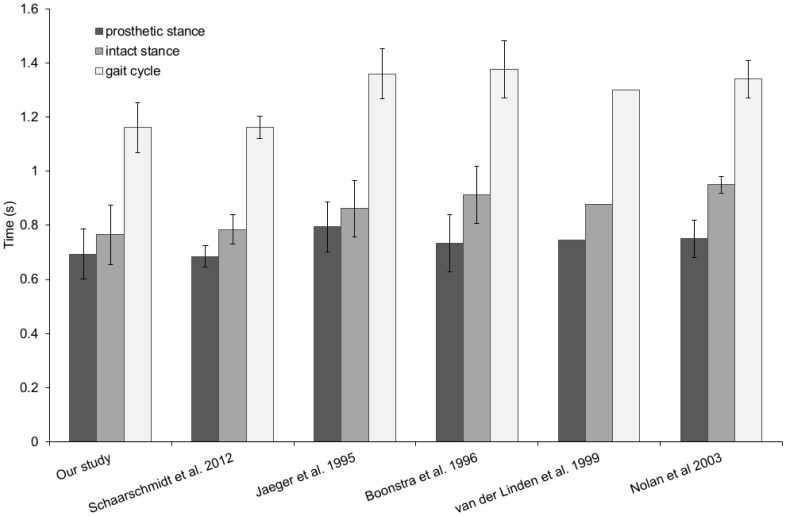
Temporal gait parameters of trans-femoral amputees level walking at self-selected speed amputees. Our YouTube study data compared against typical values obtained from the literature. Overall gait cycle time is typically around 1.2 to 1.4s, with stance being between 0.7 and 0.9s. Stance time on the prosthetic side is consistently shorter that than the intact side, by approximately a 0.1s. All data are means shown with standard deviation error bars. The study of van der Linden et al [18] only includes data for a single amputee, hence the lack error bars.

**Fig 2:**
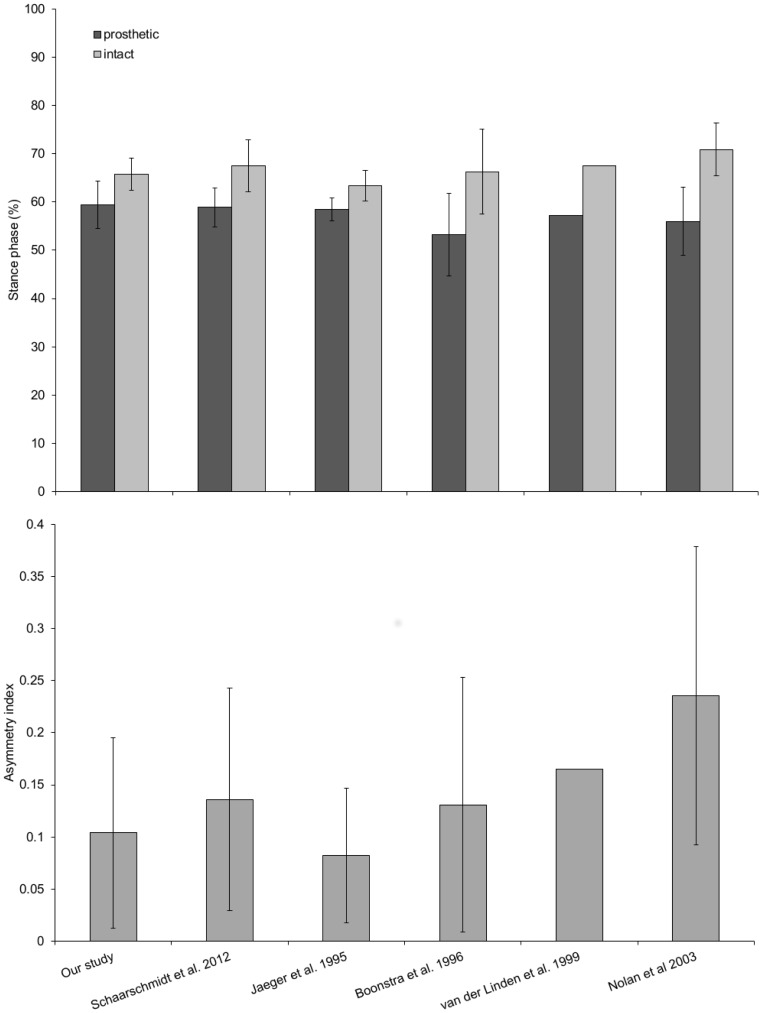
Stance phase and asymmetry index of trans-femoral amputees level walking at self-selected speed. Top: Stance phase (also known as duty factor) for both intact and prosthetic leg of unilateral trans-femoral amputees level walking at self-selected speeds. Our study results are consistent with those found in the literature with the stance phase on the prosthetic leg being consistently shorter than the intact leg. Bottom: The asymmetry index calculated from stance phase data. Our study (and literature studies) show significant asymmetry of gait for the amputees (no asymmetry would equal 0). All bars display means with standard deviation error bars. The study of van der Linden et al [18] only includes data for a single amputee, hence the lack error bars.

The results for gait cycle time, stance times, stance duty factors and asymmetry index can be seen in Table 2 and Figs 1 and 2. The mean asymmetry index (0.104), was significantly different from the null hypothesis of zero, *t*(15) = 4.55, p<0.001.

**Table 2:** Temporal gait parameters of YouTube trans-femoral amputees level walking at self-selected speed.

	Gait cycle time (s)	Intact stance time (s)	Intact stance duty factor (%)	Prosthetic stance time (s)	Prosthetic stance duty factor (%)	Asymmetry index
**Mean**	1.161	0.765	65.8	0.694	59.4	0.104
**SD**	0.111	0.111	3.3	0.093	5.0	0.091

Subjects were adult unilateral trans-femoral amputees walking on a level surface at a self-selected speed. Means and standard deviations were calculated for the sample of 16 subjects.

We used an ANOVA to compare the results of our study with data from the literature, and found some significant difference between studies (Table 3). No significant differences were found between prosthetic stance times (F(4,59) = 2.13, p = 0.088), intact stance phases (F(4,59) = 1.07, p = 0.381) or asymmetry indices (F(4,59) = 1.68, p = 0.167) from all studies. However, significant difference for intact stance time (F(4,59) = 6.92, p = 0.0001), gait cycle time (F(4,59) = 13.28 p < 0.0001) and prosthetic stance phase (F(4,59) = 2.86, p = 0.031) were found. Intact stance time from our study was significantly different from the studies of Boonstra et al. [17] and Nolan et al. [15]. No other differences in intact stance time were found. Gait cycle time for our study was significantly different from studies of Jaegar et al. [16],Boonstra et al. [17] and Nolan et al. [15] but not different from Schaarschmidt et al. [14]. Similarly, gait cycle time for Schaarschmidt et al. [14] was significantly different from Jaegar et al. [16] and Boonstra et al. [17]. No other differences in gait cycle time were found. Prosthetic stance phase for our study was significantly different from Boonstra et al. [17].

**Table 3:** Tukey-Kramer post hoc p values for intact leg stance times, gait cycle time and prosthetic stance phase.

**Intact stance time**	**Schaarschmidt et al. 2012**	**Jaeger et al. 1995**	**Boonstra et al. 1996**	**Nolan et al. 2003**
**Our study**	0.9948	0.1146	0.0002*	0.0149*
**Schaarschmidt et al. 2012**	\	0.6629	0.0837	0.1232
**Jaeger et al. 1995**	\	\	0.6264	0.5785
**Boonstra et al. 1996**	\	\	\	0.9596
**Gait cycle time**	**Schaarschmidt et al. 2012**	**Jaeger et al. 1995**	**Boonstra et al. 1996**	**Nolan et al. 2003**
**Our study**	1	0.0001*	<0.0001*	0.0332*
**Schaarschmidt et al. 2012**	\	0.0103*	0.0012*	0.1148
**Jaeger et al. 1995**	\	\	0.9918	0.9977
**Boonstra et al. 1996**	\	\	\	0.9673
**Prosthetic stance phase**	**Schaarschmidt et al. 2012**	**Jaeger et al. 1995**	**Boonstra et al. 1996**	**Nolan et al. 2003**
**Our study**	0.9999	0.9948	0.0327*	0.8836
**Schaarschmidt et al. 2012**	\	0.9999	0.4001	0.963
**Jaeger et al. 1995**	\	\	0.1968	0.9699
**Boonstra et al. 1996**	\	\	\	0.9372

p value less than 0.05 indicates a significant difference between studies (indicated with a *)

## Discussion

The use of crowd-sourced videos for temporal gait analysis in amputee locomotion has not yet been acknowledged or tested. Here we show that temporal gait data collected from YouTube videos of unilateral trans-femoral amputees is comparable and of equivalent variability to gait data published in the literature. Although we do not have access to synchronous gold standard measurements for error analysis purposes, this result lends confidence to our data collection methodology and demonstrates the potential for the future use of YouTube videos for gait data collection in general.

In this study our example dataset of temporal gait parameters for trans-femoral amputees are similar to values taken from published literature (Figs 1 and 2). Mean and standard deviation values for gait cycle time and stances times, for both intact and prosthetic side, are generally comparable to values previously published (Fig 1). Using an ANOVA differences were found for intact stance time, gait cycle time and prosthetic stance phase between some studies. The difference in gait cycle time are due to differences in cadence (steps per minute) between study subjects, which may relate to differences in walking speed (1.0 to 1.2 m/s for the cited studies included in the analysis). Differences in cadence and walking speed would also explain the difference in intact stance time and prosthetic stance phase. Difficulty in controlling (or indeed measuring) variables such as walking speed from YouTube videos is an inherent drawback of our methodology and one which needs careful consideration before embarking on studies using internet videos. Nevertheless, the similarity of our results with other published studies was surprising since we suspected that both the data collection protocol (i.e. digitising videos recorded on standard video cameras) and the uncontrolled nature of the video content (i.e. prosthesis type, gender, age etc.) may have led to an increase in the variability of the results. However, it appears that the data collection method used here produces results of a comparable variability to studies carried out under highly controlled conditions using laboratory-standard data collection tools. This suggests that either the inherent ‘noisiness’ of trans-femoral gait data is larger than the variability resulting from the differences between prosthesis types, ages etc. seen in our videos, or that in level walking trans-femoral amputees in the ‘real’ world are similar to those walking in gait labs.

The data from our YouTube study lends confidence to our methodology and suggests that the scope could be expanded in future studies. Indeed, as markerless gait analysis techniques improve (for example [19,20,21]) the potential scope for the types of data that could be collected from internet videos should increase beyond just the temporal parameters illustrated here. Furthermore whilst searching for trans-femoral walking videos for this study the number, variety and scope of potential videos found on YouTube was exceptional with many videos showing gait activities that are not normally studied, for example videos of amputees walking on rough and loose terrain. Amputee gait data for walking on anything other than flat and firm surfaces are generally quite rare in the literature, and researchers could make use of these videos to compare and contrast gait on a variety of terrains. Indeed, there are even videos of more ‘extreme’ activities such as rock climbing, snowboarding and amputee soccer, which researchers in para-sports and para-athletics may find valuable for their studies. In addition to the variety of walking surfaces and activities found in the videos, there are also videos from subjects with less prevalent amputations, such as bi-lateral trans-femoral amputations, conditions for which conventional studies are inevitably limited in terms of sample size.

Having discussed the potential merits of crowd-sourced data it is necessary to also address the significant limitations with the approach, which should be considered before future researchers engage in these types of studies. Firstly, the search strategy was limited in scope. However, it did identify a number of relevant videos, sufficient to demonstrate the feasibility of the approach. It is also interesting to note that since YouTube videos do not require the user to input structured descriptors, it remains unclear whether a significantly improved approach could be implemented and this may remain a significant limitation with the approach as presented in our paper. Furthermore YouTube videos can be uploaded or deleted by users at will (see Table 1 and video 5 of our study) and hence ensuring the repeatability of the work presents a challenge. One option which could be explored would be to make copies of the relevant videos and upload them to a more permanent location, using tools such as FigShare. However, IP, ethical and consent issues may present a major challenge to this. Secondly, considering the quality of the data, the videos are collected under typically uncontrolled lighting conditions with a range of different cameras and viewpoints. These factors might be expected to introduce error when digitising the data to derive useful parameters, such as step time and future studies may usefully consider quantifying the scale of these errors through repeatability and validity studies. For instance, the use of wearable sensors can provide “gold standard” temporal data “out of the lab” [22,23], which could be compared with data derived from video analysis. The approach, as presented in this paper, is also clearly limited by a lack of data on participants and their prosthetic components. Future studies may consider a more pro-active approach, as outlined below. Finally, it is clear that our approach could only provide limited temporal and kinematic data, even with the use of advanced image analysis tools. For research that requires more detailed data this approach is unlikely to be beneficial.

Nevertheless, the wealth of potential data available online makes the above challenges worth overcoming and we encourage this approaches consideration for future research. Indeed, both the breadth and number of amputee gait videos online is already remarkable and they are likely to continue to grow in coming years. Further, future studies could also consider taking an active ‘citizen science’ approach to data collection. For example, researchers could conduct ‘organised crowd-sourcing’ projects that ask patients with particular conditions to upload videos of themselves performing a protocol or activity. This idea could be extended from gait studies into a variety of functional tasks performed by patients. Conducting studies in this manner would help to negate some of the problems faced by current researchers, notably low statistical power. However, such an active approach would potentially create new problems in terms of co-ordination of projects, language barriers and recruitment of subjects. We encourage researchers of amputee gait to investigate the potential of our approach highlighted in this feasibility study and to also more clearly identify the scope of problems for which our technique may be suitable.

## Conclusions

The growth of easily accessible gait data on the Internet should inspire researchers to conduct studies that might have been very difficult in the pre-internet age. We have shown here that collecting an example dataset of amputee temporal gait data from YouTube videos produces results that are comparable with published data from controlled laboratory studies. Therefore, the potential for future studies to make use of YouTube and other online resources for collecting data on amputee gait is large and we encourage other researchers to explore how best to use this resource.

## Supporting Information

S1 File Excel spreadsheet containing all data and figures used in this study. The collated data contains both the data from our YouTube video analysis and the data collected from the literature studies.
(XLSX)
